# Histopathologic risk factors for progression of atypical meningioma: a retrospective cohort study evaluating the impact and clinical value of mitotic count and Ki-67

**DOI:** 10.1007/s00701-025-06711-4

**Published:** 2025-12-01

**Authors:** Yoon Hwan Byun, Mira Han, Sun Mo Nam, Jong Ha Hwang, Yong Hwy Kim, Chul-Kee Park, Min-Sung Kim

**Affiliations:** 1 Department of Neurosurgery, SMG-SNU Boramae Medical Center, Seoul, Republic of Korea; 2 Medical Research Collaborating Center, SMG-SNU Boramae Medical Center, Seoul, Republic of Korea; 3 Department of Neurosurgery, Seoul National University College of Medicine, Seoul National University Hospital, Seoul, Republic of Korea

**Keywords:** Atypical meningioma, Recurrence, Progression-free survival, Ki-67, Mitotic count, Radiotherapy

## Abstract

**Purpose:**

Given the heterogeneity of atypical meningioma (AM) and potential interobserver variability in WHO grade assignment among pathologists, there is a need for more objective criteria to improve risk stratification. This study examined conventional and novel risk factors for AM progression, focusing on mitotic count (MC) and Ki-67, and explored their clinical relevance.

**Methods:**

This retrospective cohort study included 240 consecutive patients with AM surgically treated at a single tertiary institution between 2001 and 2020. The cut-off values for MC and Ki-67 were determined using the Youden index. Risk factors for progression were analyzed using cause-specific Cox proportional hazards models. Progression-free survival (PFS) was estimated using cumulative incidence function (CIF) and compared using the Gray’s test.

**Results:**

AM progression occurred in 32.5% of patients with a median time to progression of 25.2 months. The median follow-up was 42.3 months. While a clinically meaningful Ki-67 cut-off was not identified, MC ≥ 6 was significantly associated with AM progression. On multivariate analysis, age, gross total resection (GTR), MC ≥ 6, brain invasion, sheeting, and adjuvant radiotherapy (RTx) were associated with progression. RTx improved PFS in the subtotal resection (STR) group but not in the GTR group. Among GTR patients, those with MC ≥ 6 had worse outcomes.

**Conclusion:**

GTR and RTx may reduce the progression of AM. MC ≥ 6 significantly increases the risk of progression, even in GTR patients. RTx should be considered for all STR patients A more vigilant follow-up or consideration of RTx is warranted in GTR patients when a high MC is identified.

**Supplementary Information:**

The online version contains supplementary material available at 10.1007/s00701-025-06711-4.

## Introduction

Meningiomas are the most common primary central nervous system tumors accounting for 41.7%–42.9% of cases [9, 44]. Atypical meningiomas (AM) are WHO grade 2 tumors representing 15%–20% of all meningiomas [58] with the incidence of AM reported to have increased in recent years [45]. The diagnostic criteria for AM have evolved over time [8] with a significant change in 2016 when brain invasion was introduced as one of the diagnostic histopathologic features [33]. The latest 2021 WHO classification defines AM by one or more of the following criteria: a mitotic count (MC) of 4–19/10 in 10 consecutive high-power field (HPF) of each 0.16 mm^2^, brain invasion, chordoid or clear cell histology, or at least three of five histologic features—spontaneous or geographic necrosis, prominent nucleoli, high cellularity, small cells with a high nucleus-to-cytoplasm (N/C) ratio, and sheeting [59].

AM is primarily treated with maximal safe resection, while the role of adjuvant RTx remains under investigation, particularly in cases of GTR [43]. Despite a high GTR rate, AM has a relatively high local recurrence rate of 30%–40% [1, 37] with a median time to progression of two years [7]. Recurrence of AM has been shown to be associated with morbidity, reduced survival, and additional treatment burden [27, 48]. Therefore, identifying prognostic factors for AM progression is crucial for risk stratification and treatment optimization.

Several risk factors for AM progression have been reported, with extent of resection (EOR) being one of the most important factors [10, 47]. However, previous studies exploring risk factors of AM progression have been limited by small sample sizes, short follow-up periods and mixed results [23]. Additionally, notable heterogeneity within AM and interobserver variability in WHO grading of meningiomas among pathologists [46] highlight the need for additional objective criteria, such as refined MC thresholds and the Ki-67 index, to improve risk stratification. This study aims to evaluate risk factors for AM progression, with a particular focus on MC and Ki-67, and to explore the potential clinical implications of these findings for improving patient outcomes.

## Materials and methods

### Study design

This was a retrospective cohort study of AM patients who underwent primary surgical resection between 2001 and 2022 at a single tertiary institution in the Republic of Korea (Seoul National University Hospital). A total of 323 consecutive AM cases were identified, of which 83 were excluded due to missing follow-up brain imaging. Consequently, 240 AM patients were included in the final cohort. No formal sample size calculation was performed and all eligible patients were included to maximize statistical power. All pathological reports were reviewed to confirm that the included cases met the latest 2021 WHO diagnostic criteria for AM. Clinical and histopathological variables were collected from electronic medical records, pathology reports and radiology archives. This study was approved by the Institutional Review Board of Seoul National University Hospital (IRB: 2505–039–1638).

### Clinical and histopathological variables

Clinical variables assessed included age, sex, tumor size (maximum diameter on preoperative MRI), and tumor location (convexity, falx/parasagittal, skull base, or other). The EOR was categorized as either gross total resection (GTR) or subtotal resection (STR). GTR was defined as complete macroscopic tumor removal with or without resection/coagulation of dural attachment or abnormal extradural extensions, consistent with Simpson grades 1–3 [54]. STR was defined by residual enhancing tumor on immediate postoperative MRI taken within 48 h of surgery. Postoperative MRI and operative records were collectively reviewed to ensure accurate categorization.

As the cohort spanned 21 years, temporal variation was considered. Surgery period was categorized into two groups (2001–2011 and 2012–2022), and baseline characteristics and AM progression rate were compared between the two groups. Surgery period was also included as a covariate in the cause-specific Cox regression model to account for potential temporal effects.

Histopathologic features defining AM, including brain invasion, spontaneous necrosis, prominent nucleoli, high cellularity, small cells with a high N/C ratio, and sheeting were assessed by neuro-pathologists. Brain invasion was defined as irregular, tongue-like protrusions of tumor cells into underlying parenchyma without intervening leptomeninges. Spontaneous necrosis was identified when necrotic foci were clearly separated from viable tumor tissue. Prominent nucleoli were those visible under a 10 × objective lens in ≥ 50% of the sample. High cellularity was defined as more than 53 nuclei per HPF (0.16 mm^2^). Small cells were identified as tumor cells with lymphocyte-like morphology. Sheeting was defined as the absence of whorls, lobules, syncytia, or small aggregates in at least 50% of the sample. MC was defined as the number of mitotic figures per 10 consecutive HPF of each 0.16 mm^2^ in the most mitotically active area. Phosphohistone-H3 (PHH3) immunostaining (1:100, Cell Marque, Rocklin, CA, USA) was used to distinguish mitotic figures in some problematic situations to ensure accurate measurement of MC. The Ki-67 proliferation index (1:100, mAb MIB-1; Dako, Glostrup, Denmark) was quantified using an automated cell counting algorithm on a Sectra IDS7 viewer (Sectra AB, Linköping, Sweden) from virtually scanned slides (Aperio AT2; Leica Biosystems, Wetzlar, Germany).

### Follow up and RTx

All patients underwent postoperative MRI within 48 h of surgery. Those without immediate postoperative imaging were excluded from the cohort, as the EOR could not be accurately assessed. Active surveillance was performed with serial MRI at approximately 6–12-month intervals, with shorter intervals implemented as needed, particularly in cases of STR. Tumor progression was defined as local recurrence of any size in the GTR group. In the STR group, it was defined using the RECIST (Response Evaluation Criteria in Solid Tumors) criteria [17] as an interval increase of more than 20% in the residual tumor size. Progression-free survival (PFS) was measured from the date of surgery to radiographic recurrence or was censored at the last follow-up imaging if no recurrence was observed.

RTx was defined as treatment within one year of surgery in the absence of tumor progression. The decision to use RTx, including gamma knife radiosurgery (GKS) or conventional fractionated radiotherapy, was based on the EOR and histopathologic findings determined by the surgeon and radiation oncologist.

### Statistical analysis

Descriptive statistics were used to summarize baseline characteristics. Comparisons between categorical variables were conducted using the Chi-squared test or Fisher’s exact test as appropriate. The Shapiro–Wilk test was applied to assess the normality of continuous data. Comparisons between continuous variables were performed using either the independent t-test or the Wilcoxon rank-sum test, depending on data distribution. Univariable and multivariable cause-specific Cox proportional hazards models were fitted to evaluate risk factors for progression of AM after surgical resection. Death was treated as a competing event. Covariates associated with progression in univariable analyses were considered for the multivariable model. Backward elimination was applied to obtain the most parsimonious model, while clinically relevant variables were retained regardless of statistical significance. Model performance was evaluated using the concordance index (C-index) for discrimination and the Akaike Information Criterion (AIC) for relative fit. All Cox models included RTx as a time-dependent covariate to account for the influence of RTx timing on AM progression.

The proportional hazards assumption was verified using Schoenfeld residuals. Cumulative incidence functions (CIFs) for progression and death without progression were estimated, and group comparisons were performed with Gray’s test. Analyses were performed using SAS version 9.4 (SAS Institute) and R version 4.3.1 (R Project for Statistical Computing). A *p-*value < 0.05 was considered statistically significant.

## Results

### Patient characteristics and clinical outcome

The demographic and clinical characteristics of the patients are summarized in Table 1. A total of 240 AM cases were included in the final cohort comprised of 143 females and 97 males with a mean age of 53.8 years (*SD*: 14 years). The median follow-up duration was 42.3 months (range: 0.8–218.8 months). The mean tumor size was 46.7 mm (*SD*: 15.6 mm), with convexity being the most common tumor location (*N* = 88, 36.7%). GTR was achieved in 175 patients.

**Table 1:** Demographic characteristics of 240 atypical meningioma patients

Variables		Total (N = 240)		No Progression (N = 162)		Progression (*N* = 78)		***p***	
Age (year)	Mean ± SD	53.8	± 14.0	52.6	± 13.5	56.3	± 14.8	0.057	^3)^
Sex	Female	143	59.6%	97	59.9%	46	59.0%	0.894	^1)^
	Male	97	40.4%	65	40.1%	32	41.0%		
Tumor size (mm)	Mean ± SD	46.7	± 15.6	44.8	± 16.2	50.6	± 13.5	**0.007**	^3)^
Tumor location	Convexity	88	36.7%	67	41.4%	21	26.9%	0.123	^1)^
	Falx/Parasagittal	73	30.4%	47	29.0%	26	33.3%		
	Skull base	68	28.3%	40	24.7%	28	35.9%		
	Others	11	4.6%	8	4.9%	3	3.8%		
Extent of resection	GTR	175	72.9%	131	80.9%	44	56.4%	**0.0001**	^1)^
	STR	65	27.1%	31	19.1%	34	43.6%		
Mitotic count	Mean ± SD	6.4	± 3.3	5.9	± 3.1	7.5	± 3.6	** < 0.0001**	^4)^
Brain invasion	Absent	185	77.1%	129	79.6%	56	71.8%	0.176	^1)^
	Present	55	22.9%	33	20.4%	22	28.2%		
Increased cellularity	Absent	9	38.0%	7	4.3%	2	2.6%	0.722	^2)^
	Present	231	96.3%	155	95.7%	76	97.4%		
Small cells with high N/C ratio	Absent	142	59.2%	98	60.5%	44	56.4%	0.547	^1)^
	Present	98	40.8%	64	39.5%	34	43.6%		
Prominent nucleoli	Absent	111	46.3%	76	46.9%	35	44.9%	0.766	^1)^
	Present	129	53.8%	86	53.1%	43	55.1%		
Sheeting	Absent	191	79.6%	136	84.0%	55	70.5%	**0.016**	^1)^
	Present	49	20.4%	26	16.0%	23	29.5%		
Necrosis	Absent	192	80.0%	135	83.3%	57	73.1%	0.063	^1)^
	Present	48	20.0%	27	16.7%	21	26.9%		
Adjuvant radiotherapy	No	158	65.8%	104	64.2%	54	69.2%	0.441	^1)^
	Yes	82	34.2%	58	35.8%	24	30.8%		
Ki-67 (%)	Mean ± SD	8.6	± 7.3	8.5	± 7.9	8.7	± 5.7	0.126	^4)^

Boldface type indicates statistical significance

*GTR* Gross total resection, *N/C* Nucleus-to-cytoplasm, *SD* Standard deviation, *STR* Subtotal resection

^1)^ Chi-square test, ^2)^ Fisher's exact test, ^3)^ T-test, ^4)^ Wilcoxon rank sum test

Tumor progression occurred in 78 patients (32.5%), with a median time to progression of 25.2 months (range: 3.2–118.5 months). Compared to the non-progression group, the progression group had significantly larger tumors (50.6 mm vs 44.8 mm, *p* = 0.007), lower GTR rate (56.4% vs 80.9%, *p* = 0.0001) higher MC (7.5 vs 5.9, *p* < 0.0001), and a greater proportion of sheeting (29.5% vs 16%, *p* = 0.016). However, Ki-67 levels did not differ significantly between the two groups (*p* = 0.126). Of note, Ki-67 data were missing in five patients with two in the progression group and three in the non-progression group. They were subsequently excluded during the relevant analyses regarding Ki-67.

Characteristics according to surgery period, 2001–2011 (*N* = 73) and 2012–2022 (N = 167), are summarized in Supplementary Table 1. Patients in the 2012–2022 group had a significantly higher mitotic count (6.7 ± 3.5 vs. 5.8 ± 2.8, *p* = 0.049) and a higher Ki-67 level (10.2 ± 7.8 vs. 4.7 ± 3.4, *p* < 0.0001). However, progression rates did not differ significantly between the groups (66.5% for 2012–2022 vs. 69.9% for 2001–2011, *p* = 0.605).

### Mitotic count and Ki-67 cut off level

**Fig. 1:**
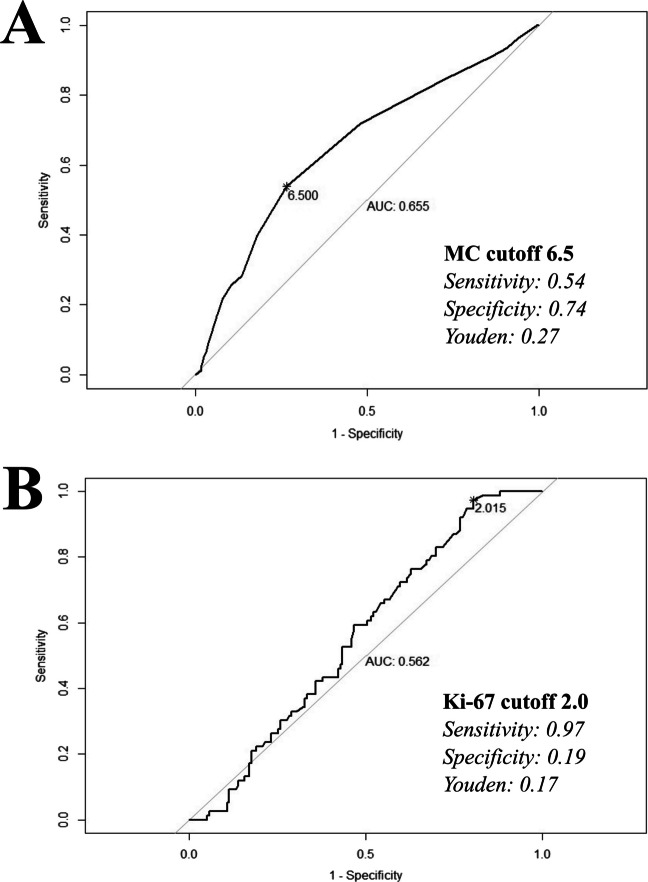
Cutoff levels for mitotic count and Ki-67. **A** Appropriate cutoff level for mitotic count (MC) associated with atypical meningioma progression was 6.5 (**B**) Ki-67 cutoff level was 2.0 which was not considered clinically significant. AUC: area under the curve; MC: mitotic count

The mean MC of the cohort was 6.4 (*SD*: 3.3), with a calculated cutoff for AM progression of 6.5 (AUC: 0.66, Youden index: 0.27). When subcategorized by EOR, the MC cutoff remained 6.5 (AUC: 0.65, Youden index: 0.31) in GTR group, while in the STR group, it was lower at 5.5 (AUC: 0.7, Youden index: 0.35). To evaluate for a clinically significant MC cutoff and adopt a more conservative approach, we conducted further risk analyses using a MC cutoff of 6, based on the overall cutoff level of 6.5. A clinically relevant Ki-67 cutoff for AM progression could not be determined, as the optimal Ki-67 cutoff in our cohort was calculated to be 2, with a low Youden index of 0.17, and an insignificant AUC of 0.56. Given that the mean Ki-67 level in the cohort was 8.6 (*SD*: 7.3), this cutoff was not considered clinically significant. (Fig. 1).

### Risk factors for AM progression

Univariate cause-specific Cox regression analysis found age (*p* = 0.006), tumor size (*p* = 0.006), surgery period (*p* = 0.034), EOR (*p* = 0.001), brain invasion (*p* = 0.017), sheeting (*p* = 0.039), MC (*p* = 0.005) and Ki-67 (*p* = 0.032) as a risk factor for AM progression. On multivariate analysis, age (HR 1.03, *p* = 0.014), GTR (HR 0.18, *p* < 0.001), brain invasion (HR 2.31, *p* = 0.002), sheeting (HR 1.69, *p* = 0.027), MC ≥ 6 (HR 2.32, *p* = 0.002) and RTx (HR 0.25, *p* < 0.001) were significantly associated with progression of AM. Ki-67 was not statistically significant on multivariate analysis (Table 2).

**Table 2:** Time-dependent univariate and multivariate cox analyses of risk factors for atypical meningioma progression

Variables		Univariate				Multivariate			
		HR	95% CI		*p*	HR	95% CI		*p*
Sex (ref: Female)	Male	1.12	(0.71,	1.75)	0.66	1.05	(0.65,	1.69)	0.846
Age (continuous)		1.02	(1.01,	1.04)	**0.006**	1.03	(1.01,	1.05)	**0.014**
Tumor size (continuous)		1.02	(1.01,	1.03)	**0.006**				
Tumor location (ref: convexity)	Falx/parasagittal	1.32	(0.74,	2.35)	0.345				
	Skull base	1.59	(0.91,	2.81)	0.106				
	other	1.34	(0.40,	4.51)	0.633				
Surgery period (ref: 2001–2011)	2012–2022	1.74	(1.04,	2.89)	**0.034**				
Extent of resection (ref: STR)	GTR	0.47	(0.30,	0.74)	**0.001**	0.18	(0.10,	0.31)	** < 0.001**
Mitotic count (ref: < 6)	≥ 6	2.04	(1.24,	3.33)	**0.005**	2.32	(1.37,	3.94)	**0.002**
Brain invasion (ref: absent)	present	1.83	(1.12,	3.02)	**0.017**	2.31	(1.35,	3.96)	**0.002**
Increased cellularity (ref: absent)	present	2.14	(0.53,	8.76)	0.288				
Small cells with high N/C ratio (ref: absent)	present	1.22	(0.78,	1.92)	0.378				
Prominent nucleoli (ref: absent)	present	1.17	(0.75,	1.82)	0.500				
Sheeting (ref: absent)	present	1.67	(1.03,	2.72)	**0.039**	1.69	(1.06,	2.69)	**0.027**
Necrosis (ref: absent)	present	1.40	(0.85,	2.31)	0.190				
Ki-67 (continuous)		1.03	(1.00,	1.07)	**0.032**				
Adjuvant radiotherapy	Yes	0.90	(0.56,	1.44)	0.662	0.25	(0.13,	0.46)	** < 0.001**

Boldface type indicates statistical signficance

*CI* Confidence interval, *GTR* Gross total resection, *HR* Hazard ratio, *N/C* Nucleus-to-Cytoplasm, *Ref* Reference, *STR* Subtotal resection

### Progression free survival

**Fig. 2:**
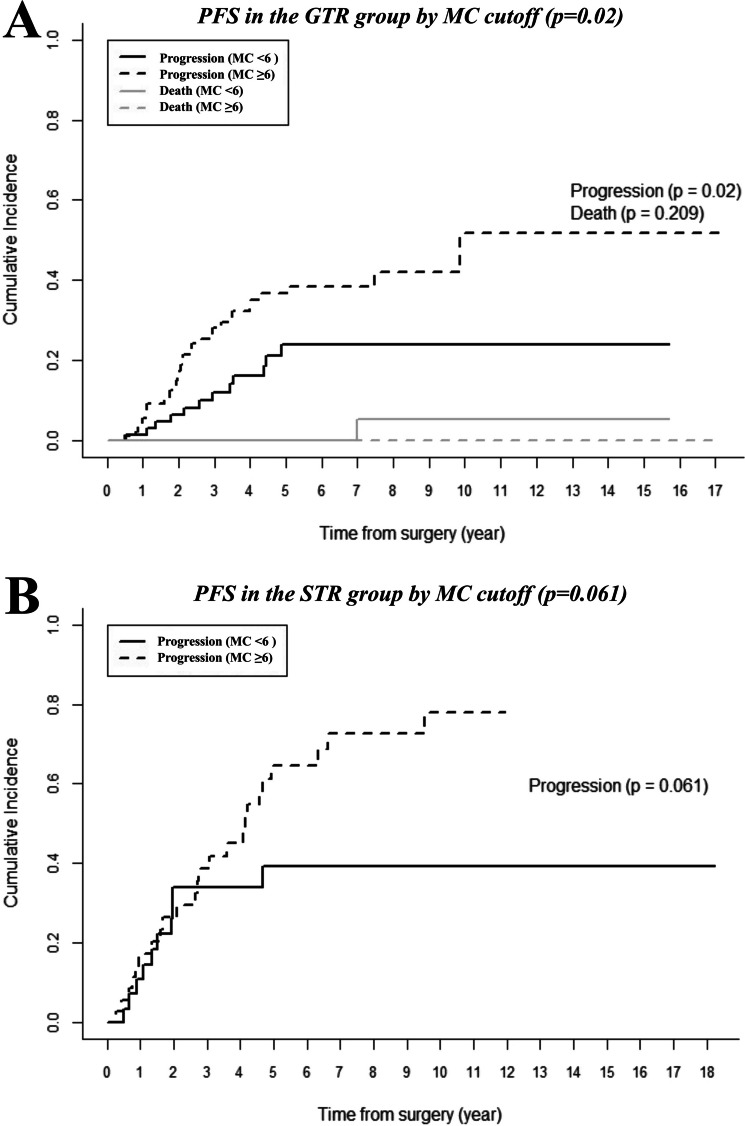
Progression-free survival (PFS) of GTR and STR groups stratified by a mitotic count (MC) cutoff of 6. **A** PFS of the GTR group. Patients with MC ≥ 6 showed significantly worse prognosis than those with MC < 6 (*p* = 0.02) (**B**) PFS of the STR group. No significant difference in PFS was observed between patients with MC ≥ 6 and MC < 6 (*p* = 0.061). PFS: progression free survival; GTR: gross total resection; MC: mitotic count; STR: subtotal resection

The GTR group had better overall PFS than the STR group (*p* = 0.001). In subgroup analyses based on a MC cutoff of 6, GTR patients with a MC ≥ 6 (*N* = 99) had a worse prognosis than those with a MC < 6 (*N* = 76) (*p* = 0.02). In the STR group, PFS did not differ significantly between patients with MC ≥ 6 and < 6 (*p* = 0.061) (Fig. 2). There was a total of three acknowledged deaths: two in the progression group and one in the non-progression group. The two deaths in the progression group were due to worsening medical conditions during hospice care following treatment for AM. The single death in the non-progression group was unrelated to AM and resulted from aspiration pneumonia secondary to progression of prostate cancer.

### Adjuvant radiotherapy (RTx)

A total of 82 patients underwent RTx, with 25 patients receiving GKS and 57 patients receiving conventional radiotherapy. The median prescription dose for GKS was 17 Gy (range: 13–25.5 Gy) delivered in one to three fractions. Conventional fractionated radiotherapy was administered at a median dose of 60 Gy (range: 45–61.2 Gy) in 10 to 33 fractions. Among 175 GTR patients, 32 (18.3%) underwent RTx, compared to 50 of 65 STR patients (76.9%) (*p* < 0.0001).

**Fig. 3:**
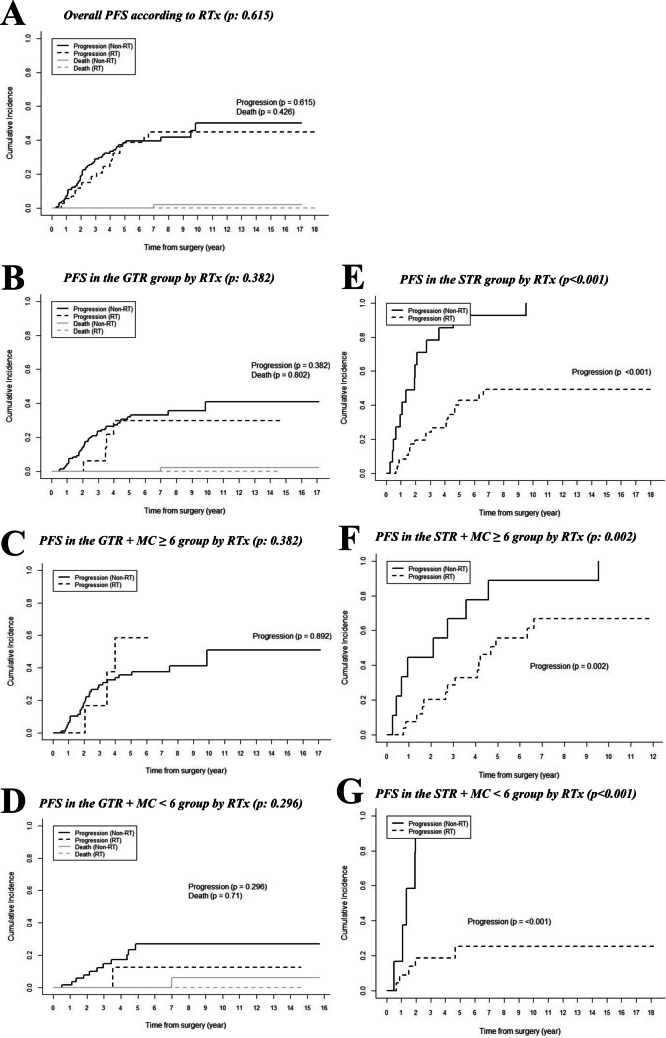
Progression-free survival (PFS) of atypical meningioma patients according to adjuvant radiotherapy (RTx), stratified by extent of resection (EOR) and mitotic count (MC) cutoff of 6. **A** Overall PFS by RTx (**B**) PFS in the GTR group (**C**) PFS in GTR with MC ≥ 6 (**D**) PFS in GTR with MC < 6 (**E**) PFS in the STR group (**F**) PFS in STR with MC ≥ 6 (**G**) PFS in STR with MC < 6. PFS did not differ between patients who received RTx and those who did not (A, *p* = 0.615). RTx had no significant impact on PFS in the GTR group (B, *p* = 0.382). In the GTR group, RTx remained statistically insignificant after sub-stratification by the MC cutoff (C, *p* = 0.892; D, *p* = 0.296).RTx was associated with improved PFS in the STR group (E, *p* < 0.001). Those who underwent RTx continued to show improved PFS in the STR group even after sub-stratification by the MC cutoff (F, *p* = 0.002; G, *p* < 0.001). EOR: extent of resection; GTR: gross total resection; MC: mitotic count; PFS: progression-free survival; RTx: adjuvant radiotherapy STR: subtotal resection

The overall PFS did not differ between AM patients who received RTx and those who did not (*p* = 0.615) (Fig. 3A). The impact of RTx was further analyzed based on a MC cutoff of 6, EOR, and when each EOR group was further sub-stratified by the MC cutoff. RTx had no statistically significant impact on PFS in either the MC ≥ 6 group (*p* = 0.541) or the MC < 6 group (*p* = 0.252). Similarly, RTx had no significant effect on PFS in the GTR group (*p* = 0.382) (Fig. 3B) and remained statistically insignificant when the GTR group was sub-stratified by the MC cutoff (Fig. 3C, 3D). However, RTx was associated with significantly better PFS in the STR group (*p* < 0.001) (Fig. 3E), and this finding remained consistent after sub-stratification by the MC cutoff (Fig. 3F, 3G).

## Discussion

The overall AM progression rate in our study was 32.5% with a median time to recurrence of 25.2 months, which were compatible with results from previous studies [7]. Age, brain invasion, sheeting, and MC were positively associated with AM progression, while GTR and RTx were identified as risk-reducing factors.

### Age and EOR

Age has been reported as one of the possible risk factors for AM progression [16, 40] with older aged AM showing worse overall survival [61]. EOR has been recognized as an important risk factor for AM progression [19, 49, 51], which aligns with the findings of our study. This supports the fundamental principle that maximal safe resection should be performed in AM whenever possible.

Our study categorized EOR into either GTR or STR, and did not stratify further according to Simpson grades [54]. Although, Simpson grades are still widely used by neurosurgeons, their role in risk stratification especially within the GTR group has been called into question [53]. Further investigation into risk stratification based on Simpson grades is warranted. However, it remains reasonable to support our institution’s practice of resecting or coagulating the involved dura and hyperostotic bone during surgical treatment of AM to achieve “maximal” resection whenever it does not significantly increase the risk of neurological deficits or wound complications.

### Minor histopathologic features

Among the five minor histopathologic features defining AM, sheeting was the only feature significantly associated with progression. The current diagnostic approach of using histopathologic features is based largely on a single-institution study [42] and its prognostic validity has since been questioned. Concerns include possible lack of prognostic relevance [3], risk of over-grading [2], and low interobserver concordance [46] raising doubts about the reliability of using these histopathologic features for AM diagnosis and risk stratification. Although literature on this issue remains limited, Lee et al. [29] reported sheeting as a potential risk factor. Chiba et al. [12] found sheeting to be associated with malignant transformation in benign meningioma, whereas other studies have reported conflicting results [34, 36]. Overall, which histopathologic features are most predictive of recurrence remains open for question.

### Brain invasion

Brain invasion has long been suggested as a possible risk factor for meningioma recurrence, but it was not until 2016 that it was incorporated as a stand-alone WHO diagnostic criterion for AM [33]. Inclusion of brain invasion into the WHO grading criteria has contributed to an increased incidence of AM [35], further adding to the heterogeneity of the group. The validity of brain invasion as an isolated diagnostic marker has been questioned [31], and there are concerns over poor interobserver reproducibility and mixed findings regarding its association with AM progression [5, 6]. However, multiple studies have demonstrated brain invasion to be in fact associated with AM progression [22, 57], particularly when accompanied with other atypical features such as a high MC. Our findings are consistent with these observations.

Brain invasion remains an intuitive and clinically relevant marker of aggressive behavior in AM. One might argue that its presence could indirectly contribute to progression by limiting the EOR. However, in our study, brain invasion remained a significant independent risk factor even after adjusting for EOR in multivariate analysis, stressing its prognostic importance. Barresi et al. [3] investigated which histopathologic features defining AM most strongly predict prognosis and found that the copresence of brain invasion, high MC, and sheeting was most predictive of early recurrence, findings that are in line with our results. While brain invasion alone may not be the most optimal predictor of recurrence, its presence alongside a high MC may help identify patients with higher risk of AM recurrence.

### Optimal mitotic count cutoff & Ki-67

The optimal MC cutoff was identified as 6.5 in our cohort. However, since MC is clinically reported as an integer, representing the number of mitotic figures in 10 consecutive HPFs, we proceeded the analyses with a cutoff of 6. This conservative threshold was chosen to explore the possible MC cutoff that had real-clinical clinical applicability and prior evidence linking increased MC to AM progression.

In multivariate Cox analysis, MC ≥ 6 was independently associated with AM progression, while Ki-67 was not. Although elevated Ki-67 has been reported as a potential risk factor in some studies [32, 55], our findings did not support these results. Both increased MC [38] and higher Ki-67 [41] are associated with higher-grade meningiomas, and Ki-67 index was significantly higher in cases with MC ≥ 6 compared to MC < 6 (mean 9.4 vs 7.5,* p* = 0.001) in our study, suggesting collinearity between the two indices. However, the reason only MC remained significant while Ki-67 did not is unclear and whether MC is a better predictor of AM progression than Ki-67 remains debatable.

Nevertheless, increased MC have been reported to be associated with AM recurrence [4, 15, 25, 28] and various mitotic cutoffs [7, 18, 24, 26, 30] have been proposed (Table 3). These thresholds may serve as practical, objective criteria for risk stratification, especially given the wide range of MC observed in the heterogeneous AM population [11]. However, relying solely on MC cutoff levels for risk stratification warrants caution due to potential misclassification and should be considered in conjunction with other reported risk factors.

**Table 3:** Suggested mitotic count cutoff levels in previous studies

No	Year	Study	*N*	Recurrence rate	Average time to recurrence (months)	Suggested MC cutoff	OR/HR (95% CI)	Other suggested risk factors	Note	Reference
1	2014	Kim et al	67	38.8%	61.8	MC > 8	HR 2.44 (1.27–3.60)	EOR, p16, CDK6, pRB protein, MIB(Ki-67), p53		[49]
2	2015	Klinger et al	57	44%	33	MC ≥ 4	HR 2.51 (0.94–6.69)	MIB(Ki-67)		[50]
3	2018	Budohoski et al	220	32%	24	MC > 7	OR 4.27 (1.4–12.19)	STR, parafalcine/parasagittal location, peritumoral edema, adjuvant radiotherapy		[11]
4	2020	Fioravanzo et al	200	49.5%	24	MC ≥ 6	OR 2.2 (1.1—4.1)	Male, parasagittal location, Simpson grade 3, sheeting	Only GTR patients with no adjuvant radiotherapy	[51]
5	2022	Lee et al	105	36.4%	49.4	MC > 8.5	HR 3.44 (1.30—5.59)	Tumor size, EOR, MIB(Ki-67)		[52]

*EOR* Extent of resection, *HR* Hazard ratio, *MC* Mitotic count cutoff, MIB, *OR* odd ratio, *STR* subtotal resection

### Adjuvant radiotherapy for AM patients

As AM patients with STR are more likely to receive RTx or undergo closer follow-up, current debate focuses on the management of those with GTR. Although RTx has been reported to reduce the risk of AM progression [14, 20, 50, 52, 56], its role in GTR remains controversial [13, 60]. Collinearity between EOR and RTx is evident, as most STR patients (76.9%) receive RTx. Nonetheless, RTx remained a significant risk-reducing factor even after adjusting for EOR. While overall PFS did not differ significantly between patients who received RTx and those who did not, subgroup analysis by EOR showed significantly better prognosis in the STR group receiving RTx.

Taken together, while it seems reasonable to recommend RTx for AM patients with STR, we propose using an MC cutoff of 6 to help identify GTR patients who may benefit from closer surveillance or consideration of RTx. There was no significant difference in PFS was observed among GTR patients with MC ≥ 6 based on RTx status; however, this may have been due to relatively small percentage of GTR patients receiving RTx and potential over-stratification. Nonetheless, since MC was a significant risk factor for AM progression, and GTR patients with MC ≥ 6 had worse prognosis than those with MC < 6, a MC cutoff of 6 may serve as a useful tool for postoperative risk stratification and treatment planning. Ongoing clinical trials [21, 39] are expected to provide more definitive evidence on the role of RTx in GTR, which may help refine future treatment strategies.

### Strengths and Limitations

The current study is limited by its retrospective design, which may have introduced selection bias, as patients without postoperative imaging were excluded. Generalizability of the results may also be restricted because the cohort was drawn from a single tertiary institution. Another limitation is the potential for interobserver variability in the diagnosis of AM. Although all pathology reports were reviewed to confirm that cases met the 2021 WHO diagnostic criteria for AM, variability may still have influenced the original diagnoses since the review was based on the initial pathology reports. Finally, five cases lacked Ki-67 data, but this represented a small proportion of the cohort and is unlikely to have significantly affected the overall results.

Despite these limitations, our study included a relatively large sample size with a mean follow-up duration of over 3 years. We investigated the potential risk factors for AM progression, focusing on identifying appropriate MC and Ki-67 cutoffs that may serve as objective criteria for stratifying risk within this heterogeneous pool of AM. These findings may help guide clinical decision-making, particularly in patients with GTR. While the proposed cutoffs should be interpreted with caution and considered alongside other risk factors, we suggest using an MC cutoff of 6 to identify GTR patients who may benefit from closer surveillance or consideration of RTx, and support recommending RTx for patients with STR.

## Supplementary Information

Below is the link to the electronic supplementary material.

ESM 1 Supplementary Material 1 (DOCX 19.0 KB)

## Data Availability

The data supporting the findings of this investigation are available upon reasonable request from the corresponding author.

## Abbreviations

*AIC*: Akaike Information Criterion
*AM*: Atypical meningioma
*AUC*: Area under the curve
*C-Index*: C oncordance index
*EOR*: Extent of resection
*GKS*: Gamma knife surgery
*GTR*: Gross total resection
*HPF*: High power field
*HR*: Hazard ratio
*MC*: Mitotic count
*MRI*: Magnetic resonance imaging
*N/C*: Nucleus-to-cytoplasm
*PFS*: Progression-free survival
*PHH3*: Phosphohistone-H3
*RECIST*: Response Evaluation Criteria in Solid Tumors
*RTx*: Adjuvant radiotherapy
*SD*: Standard deviation
*STR*: Subtotal resection
*WHO*: World Health Organization

## References

[1] Aghi MK, Carter BS, Cosgrove GR, Ojemann RG, Amin-Hanjani S, Martuza RL, Curry WT Jr., Barker FG (2009) Long-term recurrence rates of atypical meningiomas after gross total resection with or without postoperative adjuvant radiation. Neurosurgery 64:56–60. 10.1227/01.Neu.0000330399.55586.63 19145156 10.1227/01.NEU.0000330399.55586.63

[2] Barresi V, Caffo M (2017) Rhabdoid meningioma: grading and prognostic significance of this uncommon variant. J Neuropathol Exp Neurol 76:414–416. 10.1093/jnen/nlx022 28521039 10.1093/jnen/nlx022

[3] Barresi V, Lionti S, Caliri S, Caffo M (2018) Histopathological features to define atypical meningioma: what does really matter for prognosis? Brain Tumor Pathol 35:168–180. 10.1007/s10014-018-0318-z 29671247 10.1007/s10014-018-0318-z

[4] Barrett OC, Hackney JR, McDonald AM, Willey CD, Bredel M, Fiveash JB (2019) Pathologic predictors of local recurrence in atypical meningiomas following gross total resection. Int J Radiat Oncol Biol Phys 103:453–459. 10.1016/j.ijrobp.2018.09.019 30253235 10.1016/j.ijrobp.2018.09.019

[5] Behling F, Hempel JM, Schittenhelm J (2021) Brain invasion in meningioma-a prognostic potential worth exploring. Cancers (Basel). 10.3390/cancers13133259 34209798 10.3390/cancers13133259 PMC8267840

[6] Brokinkel B, Hess K, Mawrin C (2017) Brain invasion in meningiomas-clinical considerations and impact of neuropathological evaluation: a systematic review. Neuro Oncol 19:1298–1307. 10.1093/neuonc/nox071 28419308 10.1093/neuonc/nox071 PMC5596167

[7] Budohoski KP, Clerkin J, Millward CP, O’Halloran PJ, Waqar M, Looby S, Young AMH, Guilfoyle MR, Fitzroll D, Devadass A, Allinson K, Farrell M, Javadpour M, Jenkinson MD, Santarius T, Kirollos RW (2018) Predictors of early progression of surgically treated atypical meningiomas. Acta Neurochir (Wien) 160:1813–1822. 10.1007/s00701-018-3593-x 29961125 10.1007/s00701-018-3593-x PMC6105233

[8] Bulleid LS, James Z, Lammie A, Hayhurst C, Leach PA (2020) The effect of the revised WHO classification on the incidence of grade II meningioma. Br J Neurosurg 34:584–586. 10.1080/02688697.2019.1639616 31284782 10.1080/02688697.2019.1639616

[9] Byun YH, Ha J, Kang H, Park CK, Jung KW, Yoo H (2024) Changes in the epidemiologic pattern of primary CNS tumors in response to the aging population: an updated nationwide cancer registry data in the Republic of Korea. JCO Glob Oncol 10:e2300352. 10.1200/GO.23.00352 38301181 10.1200/GO.23.00352 PMC10846785

[10] Champeaux C, Dunn L (2016) World Health Organization grade II meningioma: a 10-year retrospective study for recurrence and prognostic factor assessment. World Neurosurg 89:180–186. 10.1016/j.wneu.2016.01.055 26850975 10.1016/j.wneu.2016.01.055

[11] Chen WC, Magill ST, Wu A, Vasudevan HN, Morin O, Aghi MK, Theodosopoulos PV, Perry A, McDermott MW, Sneed PK, Braunstein SE, Raleigh DR (2018) Histopathological features predictive of local control of atypical meningioma after surgery and adjuvant radiotherapy. J Neurosurg 130:443–450. 10.3171/2017.9.JNS171609 29624151 10.3171/2017.9.JNS171609

[12] Chiba K, Sugawara T, Kobayashi D, Sato A, Murota Y, Maehara T (2021) Atypical histological features as risk factors for recurrence in newly diagnosed WHO grade I meningioma. Neurol Med Chir (Tokyo) 61:647–651. 10.2176/nmc.oa.2021-0153 34470989 10.2176/nmc.oa.2021-0153 PMC8592816

[13] Chun SW, Kim KM, Kim MS, Kang H, Dho YS, Seo Y, Kim JW, Kim YH, Park CK (2021) Adjuvant radiotherapy versus observation following gross total resection for atypical meningioma: a systematic review and meta-analysis. Radiat Oncol 16:34. 10.1186/s13014-021-01759-9 33596974 10.1186/s13014-021-01759-9 PMC7890913

[14] Dobran M, Marini A, Splavski B, Rotim K, Liverotti V, Nasi D, Iacoangeli M (2020) Surgical treatment and predictive factors for atypical meningiomas: a multicentric experience. World Neurosurg 144:e1–e8. 10.1016/j.wneu.2020.03.201 32311549 10.1016/j.wneu.2020.03.201

[15] Domingo RA, Tripathi S, Vivas-Buitrago T, Lu VM, Chaichana KL, Quinones-Hinojosa A (2020) Mitotic index and progression-free survival in atypical meningiomas. World Neurosurg 142:191–196. 10.1016/j.wneu.2020.06.189 32615290 10.1016/j.wneu.2020.06.189

[16] Durand A, Labrousse F, Jouvet A, Bauchet L, Kalamaridès M, Menei P, Deruty R, Moreau JJ, Fèvre-Montange M, Guyotat J (2009) WHO grade II and III meningiomas: a study of prognostic factors. J Neurooncol 95:367–375. 10.1007/s11060-009-9934-0 19562258 10.1007/s11060-009-9934-0

[17] Eisenhauer EA, Therasse P, Bogaerts J, Schwartz LH, Sargent D, Ford R, Dancey J, Arbuck S, Gwyther S, Mooney M, Rubinstein L, Shankar L, Dodd L, Kaplan R, Lacombe D, Verweij J (2009) New response evaluation criteria in solid tumours: revised RECIST guideline (version 1.1). Eur J Cancer 45:228–247. 10.1016/j.ejca.2008.10.026 19097774 10.1016/j.ejca.2008.10.026

[18] Fioravanzo A, Caffo M, Di Bonaventura R, Gardiman MP, Ghimenton C, Ius T, Maffeis V, Martini M, Nicolato A, Pallini R, Pegolo E, Pinna G, Sala F, Skrap M, Volpin V, Barresi V (2020) A risk score based on 5 clinico-pathological variables predicts recurrence of atypical meningiomas. J Neuropathol Exp Neurol 79:500–507. 10.1093/jnen/nlaa018 32232472 10.1093/jnen/nlaa018

[19] Goyal LK, Suh JH, Mohan DS, Prayson RA, Lee J, Barnett GH (2000) Local control and overall survival in atypical meningioma: a retrospective study. Int J Radiat Oncol Biol Phys 46:57–61. 10.1016/s0360-3016(99)00349-1 10656373 10.1016/s0360-3016(99)00349-1

[20] Hasan S, Young M, Albert T, Shah AH, Okoye C, Bregy A, Lo SS, Ishkanian F, Komotar RJ (2015) The role of adjuvant radiotherapy after gross total resection of atypical meningiomas. World Neurosurg 83:808–815. 10.1016/j.wneu.2014.12.037 25535067 10.1016/j.wneu.2014.12.037

[21] Jenkinson MD, Javadpour M, Haylock BJ, Young B, Gillard H, Vinten J, Bulbeck H, Das K, Farrell M, Looby S, Hickey H, Preusser M, Mallucci CL, Hughes D, Gamble C, Weber DC (2015) The ROAM/EORTC-1308 trial: radiation versus observation following surgical resection of atypical meningioma: study protocol for a randomised controlled trial. Trials 16:519. 10.1186/s13063-015-1040-3 26576533 10.1186/s13063-015-1040-3 PMC4650615

[22] Karabagli P, Karabagli H, Mavi Z, Demir F, Ozkeles EY (2020) Histopathological and clinical features as prognostic factors of atypical meningiomas. Turk Neurosurg 30:575–746. 10.5137/1019-5149.JTN.31161-20.1 10.5137/1019-5149.JTN.31161-20.1 32705671

[23] Kim MS, Chun SW, Dho YS, Seo Y, Lee JH, Won JK, Kim JW, Park CK, Park SH, Kim YH (2022) Histopathological predictors of progression-free survival in atypical meningioma: a single-center retrospective cohort and meta-analysis. Brain Tumor Pathol 39:99–110. 10.1007/s10014-021-00419-w 35031884 10.1007/s10014-021-00419-w

[24] Kim MS, Kim KH, Lee EH, Lee YM, Lee SH, Kim HD, Kim YZ (2014) Results of immunohistochemical staining for cell cycle regulators predict the recurrence of atypical meningiomas. J Neurosurg 121:1189–1200. 10.3171/2014.7.JNS132661 25148008 10.3171/2014.7.JNS132661

[25] Kim YJ, Ketter R, Steudel WI, Feiden W (2007) Prognostic significance of the mitotic index using the mitosis marker anti-phosphohistone H3 in meningiomas. Am J Clin Pathol 128:118–125. 10.1309/HXUNAG34B3CEFDU8 17580279 10.1309/HXUNAG34B3CEFDU8

[26] Klinger DR, Flores BC, Lewis JJ, Hatanpaa K, Choe K, Mickey B, Barnett S (2015) Atypical meningiomas: recurrence, reoperation, and radiotherapy. World Neurosurg 84:839–845. 10.1016/j.wneu.2015.04.033 25916182 10.1016/j.wneu.2015.04.033

[27] Komotar RJ, Iorgulescu JB, Raper DM, Holland EC, Beal K, Bilsky MH, Brennan CW, Tabar V, Sherman JH, Yamada Y, Gutin PH (2012) The role of radiotherapy following gross-total resection of atypical meningiomas. J Neurosurg 117:679–686. 10.3171/2012.7.Jns112113 22920955 10.3171/2012.7.JNS112113

[28] Kwon SM, Kim JH, Kim YH, Hong SH, Cho YH, Kim CJ, Nam SJ (2022) Clinical implications of the mitotic index as a predictive factor for malignant transformation of atypical meningiomas. J Korean Neurosurg Soc 65:297–306. 10.3340/jkns.2021.0114 34879641 10.3340/jkns.2021.0114 PMC8918253

[29] Lee KD, DePowell JJ, Air EL, Dwivedi AK, Kendler A, McPherson CM (2013) Atypical meningiomas: is postoperative radiotherapy indicated? Neurosurg Focus 35:E15. 10.3171/2013.9.FOCUS13325 24289123 10.3171/2013.9.FOCUS13325

[30] Lee SH, Lee EH, Sung KS, Kim DC, Kim YZ, Song YJ (2022) Ki67 index is the most powerful factor for predicting the recurrence in atypical meningioma: retrospective analysis of 99 patients in two institutes. J Korean Neurosurg Soc 65:558–571. 10.3340/jkns.2021.0196 35418005 10.3340/jkns.2021.0196 PMC9271814

[31] Li HY, Ying YZ, Zheng D, Dong GH, Zhang GB, Liu XM, Lin S, Ren XH, Jiang ZL (2023) Is brain invasion sufficient as a stand-alone criterion for grading atypical meningioma? J Neurosurg 139:953–964. 10.3171/2023.2.JNS222751 37561905 10.3171/2023.2.JNS222751

[32] Liu N, Song SY, Jiang JB, Wang TJ, Yan CX (2020) The prognostic role of Ki-67/MIB-1 in meningioma: a systematic review with meta-analysis. Medicine (Baltimore) 99:e18644. 10.1097/MD.0000000000018644 32118704 10.1097/MD.0000000000018644 PMC7478528

[33] Louis DN, Perry A, Reifenberger G, von Deimling A, Figarella-Branger D, Cavenee WK, Ohgaki H, Wiestler OD, Kleihues P, Ellison DW (2016) The 2016 World Health Organization classification of tumors of the central nervous system: a summary. Acta Neuropathol 131:803–820. 10.1007/s00401-016-1545-1 27157931 10.1007/s00401-016-1545-1

[34] Marciscano AE, Stemmer-Rachamimov AO, Niemierko A, Larvie M, Curry WT, Barker FG 2nd, Martuza RL, McGuone D, Oh KS, Loeffler JS, Shih HA (2016) Benign meningiomas (WHO grade I) with atypical histological features: correlation of histopathological features with clinical outcomes. J Neurosurg 124:106–114. 10.3171/2015.1.Jns142228 26274991 10.3171/2015.1.JNS142228

[35] Messerer M, Richoz B, Cossu G, Dhermain F, Hottinger AF, Parker F, Levivier M, Daniel RT (2016) Recent advances in the management of atypical meningiomas. Neurochirurgie 62:213–222. 10.1016/j.neuchi.2016.02.003 27370103 10.1016/j.neuchi.2016.02.003

[36] Nakasu S, Fukami T, Jito J, Nozaki K (2009) Recurrence and regrowth of benign meningiomas. Brain Tumor Pathol 26:69–72. 10.1007/s10014-009-0251-2 19856217 10.1007/s10014-009-0251-2

[37] Nanda A, Bir SC, Konar S, Maiti T, Kalakoti P, Jacobsohn JA, Guthikonda B (2016) Outcome of resection of WHO grade II meningioma and correlation of pathological and radiological predictive factors for recurrence. J Clin Neurosci 31:112–121. 10.1016/j.jocn.2016.02.021 27427214 10.1016/j.jocn.2016.02.021

[38] Olar A, Wani KM, Sulman EP, Mansouri A, Zadeh G, Wilson CD, DeMonte F, Fuller GN, Aldape KD (2015) Mitotic index is an independent predictor of recurrence-free survival in meningioma. Brain Pathol 25:266–275. 10.1111/bpa.12174 25040885 10.1111/bpa.12174 PMC4297750

[39] Oncology N (2017) Phase III trial (NRG-BN003) of observation versus irradiation for a gross totally resected grade II meningioma. *ClinicalTrials.gov.* https://clinicaltrials.gov/ct2/show/NCT03180268. Accessed 22 Apr 2025

[40] Pasquier D, Bijmolt S, Veninga T, Rezvoy N, Villa S, Krengli M, Weber DC, Baumert BG, Canyilmaz E, Yalman D, Szutowicz E, Tzuk-Shina T, Mirimanoff RO, Rare Cancer N (2008) Atypical and malignant meningioma: outcome and prognostic factors in 119 irradiated patients. A multicenter, retrospective study of the Rare Cancer Network. Int J Radiat Oncol Biol Phys 71:1388–1393. 10.1016/j.ijrobp.2007.12.020 18294779 10.1016/j.ijrobp.2007.12.020

[41] Pavelin S, Becic K, Forempoher G, Mrklic I, Pogorelic Z, Titlic M, Andelinovic S (2013) Expression of Ki-67 and p53 in meningiomas. Neoplasma 60:480–485. 10.4149/neo_2013_062 23790165 10.4149/neo_2013_062

[42] Perry A, Stafford SL, Scheithauer BW, Suman VJ, Lohse CM (1997) Meningioma grading: an analysis of histologic parameters. Am J Surg Pathol 21:1455–1465. 10.1097/00000478-199712000-00008 9414189 10.1097/00000478-199712000-00008

[43] Poulen G, Vignes JR, Le Corre M, Loiseau H, Bauchet L (2020) WHO grade II meningioma: epidemiology, survival and contribution of postoperative radiotherapy in a multicenter cohort of 88 patients. Neurochirurgie 66:73–79. 10.1016/j.neuchi.2019.12.008 32145249 10.1016/j.neuchi.2019.12.008

[44] Price M, Ballard C, Benedetti J, Neff C, Cioffi G, Waite KA, Kruchko C, Barnholtz-Sloan JS, Ostrom QT (2024) CBTRUS statistical report: primary brain and other central nervous system tumors diagnosed in the United States in 2017–2021. Neuro Oncol 26:vi1–vi85. 10.1093/neuonc/noae145 39371035 10.1093/neuonc/noae145 PMC11456825

[45] Recker MJ, Kuo CC, Prasad D, Attwood K, Plunkett RJ (2022) Incidence trends and survival analysis of atypical meningiomas: a population-based study from 2004 to 2018. J Neurooncol 160:13–22. 10.1007/s11060-022-04085-6 35819682 10.1007/s11060-022-04085-6

[46] Rogers CL, Perry A, Pugh S, Vogelbaum MA, Brachman D, McMillan W, Jenrette J, Barani I, Shrieve D, Sloan A, Bovi J, Kwok Y, Burri SH, Chao ST, Spalding AC, Anscher MS, Bloom B, Mehta M (2016) Pathology concordance levels for meningioma classification and grading in NRG oncology RTOG trial 0539. Neuro Oncol 18:565–574. 10.1093/neuonc/nov247 26493095 10.1093/neuonc/nov247 PMC4799683

[47] Rogers L, Barani I, Chamberlain M, Kaley TJ, McDermott M, Raizer J, Schiff D, Weber DC, Wen PY, Vogelbaum MA (2015) Meningiomas: knowledge base, treatment outcomes, and uncertainties. A RANO review. J Neurosurg 122:4–23. 10.3171/2014.7.JNS131644 25343186 10.3171/2014.7.JNS131644 PMC5062955

[48] Rogers L, Gilbert M, Vogelbaum MA (2010) Intracranial meningiomas of atypical (WHO grade II) histology. J Neurooncol 99:393–405. 10.1007/s11060-010-0343-1 20740303 10.1007/s11060-010-0343-1

[49] Ros-Sanjuan A, Iglesias-Morono S, Carrasco-Brenes A, Bautista-Ojeda D, Arraez-Sanchez MA (2019) Atypical meningiomas: histologic and clinical factors associated with recurrence. World Neurosurg 125:e248–e256. 10.1016/j.wneu.2019.01.056 30684705 10.1016/j.wneu.2019.01.056

[50] Rydzewski NR, Lesniak MS, Chandler JP, Kalapurakal JA, Pollom E, Tate MC, Bloch O, Kruser T, Dalal P, Sachdev S (2018) Gross total resection and adjuvant radiotherapy most significant predictors of improved survival in patients with atypical meningioma. Cancer 124:734–742. 10.1002/cncr.31088 29131312 10.1002/cncr.31088

[51] Sanikommu S, Panchawagh S, Eatz T, Lu VM, Rodrigues PB, Abdelsalam A, Gurses ME, Cummings A, Uppalapati V, Akurati S, Kondoor V, Komotar RJ, Ivan ME (2024) Recurrence of atypical and anaplastic intracranial meningiomas: a meta-analysis of risk factors. Clin Neurol Neurosurg 244:108450. 10.1016/j.clineuro.2024.108450 39018991 10.1016/j.clineuro.2024.108450

[52] Shakir SI, Souhami L, Petrecca K, Mansure JJ, Singh K, Panet-Raymond V, Shenouda G, Al-Odaini AA, Abdulkarim B, Guiot MC (2018) Prognostic factors for progression in atypical meningioma. J Neurosurg 129:1240–1248. 10.3171/2017.6.JNS17120 29350599 10.3171/2017.6.JNS17120

[53] Simon M, Gousias K (2024) Grading meningioma resections: the Simpson classification and beyond. Acta Neurochir (Wien) 166:28. 10.1007/s00701-024-05910-9 38261164 10.1007/s00701-024-05910-9 PMC10806026

[54] Simpson D (1957) The recurrence of intracranial meningiomas after surgical treatment. J Neurol Neurosurg Psychiatry 20:22–39. 10.1136/jnnp.20.1.22 13406590 10.1136/jnnp.20.1.22 PMC497230

[55] Umekawa M, Shinya Y, Hasegawa H, Morshed RA, Katano A, Shinozaki-Ushiku A, Saito N (2024) Ki-67 labeling index predicts tumor progression patterns and survival in patients with atypical meningiomas following stereotactic radiosurgery. J Neurooncol 167:51–61. 10.1007/s11060-023-04537-7 38369575 10.1007/s11060-023-04537-7 PMC10978635

[56] Unterberger A, Nguyen T, Duong C, Kondajji A, Kulinich D, Yang I (2021) Meta-analysis of adjuvant radiotherapy for intracranial atypical and malignant meningiomas. J Neurooncol 152:205–216. 10.1007/s11060-020-03674-7 33635510 10.1007/s11060-020-03674-7

[57] Vranic A, Popovic M, Cor A, Prestor B, Pizem J (2010) Mitotic count, brain invasion, and location are independent predictors of recurrence-free survival in primary atypical and malignant meningiomas: a study of 86 patients. Neurosurgery 67:1124–1132. 10.1227/NEU.0b013e3181eb95b7 20881577 10.1227/NEU.0b013e3181eb95b7

[58] Wilson TA, Huang L, Ramanathan D, Lopez-Gonzalez M, Pillai P, De Los Reyes K, Kumal M, Boling W (2020) Review of atypical and anaplastic meningiomas: classification, molecular biology, and management. Front Oncol 10:565582. 10.3389/fonc.2020.565582 33330036 10.3389/fonc.2020.565582 PMC7714950

[59] Yarabarla V, Mylarapu A, Han TJ, McGovern SL, Raza SM, Beckham TH (2023) Intracranial meningiomas: an update of the 2021 World Health Organization classifications and review of management with a focus on radiation therapy. Front Oncol 13:1137849. 10.3389/fonc.2023.1137849 37675219 10.3389/fonc.2023.1137849 PMC10477988

[60] Yoon H, Mehta MP, Perumal K, Helenowski IB, Chappell RJ, Akture E, Lin Y, Marymont MA, Sejpal S, Parsa A, Chandler JR, Bendok BR, Rosenow J, Salamat S, Kumthekar P, Raizer JK, Baskaya MK (2015) Atypical meningioma: randomized trials are required to resolve contradictory retrospective results regarding the role of adjuvant radiotherapy. J Cancer Res Ther 11:59–66. 10.4103/0973-1482.148708 25879338 10.4103/0973-1482.148708

[61] Zaher A, Abdelbari Mattar M, Zayed DH, Ellatif RA, Ashamallah SA (2013) Atypical meningioma: a study of prognostic factors. World Neurosurg 80:549–553. 10.1016/j.wneu.2013.07.001 23871812 10.1016/j.wneu.2013.07.001

