# Supplementary material for: Histopathologic risk factors for progression of atypical meningioma: a retrospective cohort study evaluating the impact and clinical value of mitotic count and Ki-67
Source: Acta Neurochir (Wien). 2025 Dec 1;167(1):303. doi: 10.1007/s00701-025-06711-4 (PMC12669296; doi:10.1007/s00701-025-06711-4)
Supplement: Supplementary file 1 — Supplementary Material 1 (DOCX 19.0 KB) [file 701_2025_6711_MOESM1_ESM.docx]

**Supplementary Table 1. Baseline Characteristics According to Surgery Period (2001-2011 vs 2012-2022)**

| **Variables** |  | **2001-2011 (N=73)** | | | **2012-2022 (N=167)** | | | | ***p*** | |  | |  |  |  |
| --- | --- | --- | --- | --- | --- | --- | --- | --- | --- | --- | --- | --- | --- | --- | --- |
| **Age (year)** | Mean ± SD | 52.1 | ±12.8 | | 54.6 | | ±14.4 | | 0.215 | | ^3)^ | |  |  |  |
| **Sex** | Female | 46 | 63% | | 97 | | 58.1% | | 0.474 | | ^1)^ | |  |  |  |
|  | Male | 27 | 37% | | 70 | | 41.9% | |  | |  | |  |  |  |
| **Tumor size (mm)** | Mean ± SD | 46 | ±15.7 | | 47 | | ±15.6 | | 0.661 | | ^3)^ | |  |  |  |
| **Tumor location** | Convexity | 25 | 34.2% | | 63 | | 37.7% | | 0.715 | | ^1)^ | |  |  |  |
|  | Falx/Parasagittal | 23 | 31.5% | | 50 | | 29.9% | |  | |  | |  |  |  |
|  | Skull base | 23 | 31.5% | | 45 | | 26.9% | |  | |  | |  |  |  |
|  | Others | 2 | 2.7% | | 9 | | 5.4% | |  | |  | |  |  |  |
| **Extent of resection** | GTR | 48 | 65.8% | | 127 | | 76% | | 0.099 | | ^1)^ | |  |  |  |
|  | STR | 25 | 34.2% | | 40 | | 24% | |  | |  | |  |  |  |
| **Mitotic count** | Mean ± SD | 5.8 | ±2.8 | | 6.7 | | ±3.5 | | **0.049** | | ^4)^ | |  |  |  |
| **Brain invasion** | Absent | 58 | 79.5% | | 127 | | 76% | | 0.564 | | ^1)^ | |  |  |  |
|  | Present | 15 | 20.5% | | 40 | | 24% | |  | |  | |  |  |  |
| **Increased cellularity** | Absent | 6 | 8.2% | | 3 | | 1.8% | | **0.025** | | ^2)^ | |  |  |  |
|  | Present | 67 | 91.8% | | 164 | | 98.2% | |  | |  | |  |  |  |
| **Small cells with high N/C ratio** | Absent | 43 | 58.9% | | 99 | | 59.3% | | 0.956 | | ^1)^ | |  |  |  |
|  | Present | 30 | 41.1% | | 68 | | 40.7% | |  | |  | |  |  |  |
| **Prominent nucleoli** | Absent | 35 | 47.9% | | 76 | | 45.5% | | 0.728 | | ^1)^ | |  |  |  |
|  | Present | 38 | 52.1% | | 91 | | 54.5% | |  | |  | |  |  |  |
| **Sheeting** | Absent | 56 | 76.7% | | 135 | | 80.8% | | 0.466 | | ^1)^ | |  |  |  |
|  | Present | 17 | 23.3% | | 32 | | 19.2% | |  | |  | |  |  |  |
| **Necrosis** | Absent | 57 | 78.1% | | 135 | | 80.8% | | 0.623 | | ^1)^ | |  |  |  |
|  | Present | 16 | 21.9% | | 32 | | 19.2% | |  | |  | |  |  |  |
| **Adjuvant radiotherapy** | No | 42 | 57.5% | | 116 | | 69.5% | | 0.073 | | ^1)^ | |  |  |  |
|  | Yes | 31 | 42.5% | | 51 | | 30.5% | |  | |  | |  |  |  |
| **ki-67 (%)** | Mean ± SD | 4.7 | ±3.4 | | 10.2 | | ±7.8 | | **<.0001** | | ^4)^ | |  |  |  |
| **Progression** | No | 51 | 69.9% | | 111 | | 66.5% | | 0.605 | | ^1)^ | |  |  |  |
|  | Yes | 22 | 30.1% | | 56 | | 33.5% | |  | |  | |  |  |  |
| Boldface type indicates statistical significance | | | |  | |  | |  | |  | |  | |  |  |
| *GTR: Gross total resection, N/C: Nucleus-to-cytoplasm, SD: Standard deviation, STR: Subtotal resection* | | | | | | | | | | | | | | |  |

^1)^ Chi-square test, ^2)^ Fisher's exact test, ^3)^ T-test, ^4)^ Wilcoxon rank sum test
